# Comparison of ERAS-based multimodal pain nursing versus conventional analgesic care on postoperative pain and recovery after total hip arthroplasty: A retrospective study

**DOI:** 10.1097/MD.0000000000046508

**Published:** 2026-01-02

**Authors:** Yumei Fang, Yixin Yu

**Affiliations:** a The Operating Room, The First People’s Hospital of Lin’an District, Hangzhou City, China.

**Keywords:** analgesic efficacy, complications, ERAS, multimodal pain nursing, rehabilitation, total hip arthroplasty

## Abstract

This study aimed to retrospectively compare the effects of enhanced recovery after surgery (ERAS)-based multimodal pain nursing with conventional analgesic care on postoperative pain, opioid use, recovery, and complications in patients undergoing total hip arthroplasty (THA). A single-center retrospective study was conducted in 120 patients who underwent unilateral THA between March 2022 and April 2025. Patients were allocated to an ERAS group (n = 60) or a control group (n = 60) according to postoperative nursing protocols. Demographic and clinical data, perioperative pain management details, pain intensity, time to first ambulation, length of hospital stay, Harris hip scores, opioid consumption within 48 hours (morphine milligram equivalents), and postoperative complications were collected. Pain intensity was assessed using the numerical rating scale at 6, 12, 24, 48, and 72 hours postoperatively. Statistical analyses were performed using SPSS 26.0. Baseline characteristics were comparable between groups (all *P* > .05). Compared with conventional care, the ERAS group had significantly lower numerical rating scale scores at 6, 12, 24, 48, and 72 hours after surgery (all *P* < .05), lower opioid consumption within 48 hours (39.6 ± 7.9 vs 52.4 ± 10.2 morphine milligram equivalents, *P* < .001), earlier ambulation (23.1 ± 5.0 vs 31.2 ± 6.3 hours, *P* < .001), shorter hospital stay (7.3 ± 1.5 vs 9.0 ± 1.7 days, *P* < .001), and higher Harris hip scores at 1 month (86.8 ± 5.5 vs 80.1 ± 6.0, *P* < .001). The overall complication rate within 3 days postoperatively was significantly lower in the ERAS group (11.7% vs 46.7%, *P* < .001), particularly for drug-related adverse effects such as nausea and vomiting. ERAS-based multimodal pain nursing provides superior analgesia, reduces opioid requirements, minimizes postoperative complications, and promotes faster functional recovery following THA. This evidence supports the integration of ERAS principles into perioperative pain management protocols to enhance recovery quality and patient safety in orthopedic nursing practice.

## 1. Introduction

Total hip arthroplasty (THA) is an effective surgical intervention for end-stage hip disorders such as avascular necrosis of the femoral head and advanced osteoarthritis, providing significant improvements in joint function and quality of life.^[1,2]^ With the rapid progression of population aging, the clinical demand for THA continues to rise, and postoperative recovery has become a key determinant of both patient well-being and functional reintegration into society. However, postoperative pain remains one of the most common and challenging problems after THA. A considerable proportion of patients experience moderate to severe pain, which, if inadequately controlled, can cause substantial physical and psychological distress and may trigger serious adverse outcomes.^[3–5]^ Unrelieved pain often contributes to anxiety, sleep disturbances, and limited mobility, delaying rehabilitation and increasing the risk of complications such as deep vein thrombosis (DVT), pulmonary infection, muscle atrophy, and joint stiffness.^[6–8]^

Conventional postoperative analgesia often relies on single-agent pharmacological strategies, particularly opioid-based regimens. While opioids can partially alleviate pain, their use is associated with limitations, including inconsistent efficacy, a high incidence of adverse drug reactions (e.g., nausea, vomiting, respiratory depression), and insufficient facilitation of early rehabilitation.^[9,10]^ Such limitations may compromise patient compliance and surgical outcomes. In recent years, the concept of enhanced recovery after surgery (ERAS) has been increasingly applied in orthopedics. Within this framework, perioperative pain management has evolved from single-agent therapy toward multimodal strategies that integrate pharmacological and non-pharmacological approaches.^[11]^ Multimodal pain nursing, guided by the ERAS philosophy of “reducing stress and promoting recovery,” combines various interventions—such as drug combinations, nerve blocks, and psychological support—to achieve multi-target, synergistic analgesia, thereby alleviating pain and facilitating early functional recovery.^[12–14]^

Although the theoretical advantages of ERAS-based multimodal analgesia are widely acknowledged, and preliminary evidence from prospective studies has supported its clinical value, its implementation in routine practice across different levels of healthcare systems in China remains heterogeneous. In particular, the role of operating room nursing in systematically standardizing and integrating multimodal analgesia into perioperative care, and translating these practices into measurable short-term patient benefits, requires further real-world validation. Many institutions continue to follow conventional postoperative analgesic pathways, potentially limiting recovery outcomes.

Therefore, systematically comparing ERAS-based multimodal pain nursing with conventional analgesic care in THA patients is of significant clinical and socioeconomic relevance. By retrospectively analyzing differences in postoperative pain intensity, opioid consumption, rehabilitation outcomes, and complication rates, this study aims to provide evidence for optimizing postoperative pain management in THA and to support the standardized implementation of ERAS principles in orthopedic perioperative nursing.

## 2. Materials and methods

### 2.1. Patients and study population

This study was approved by the Ethics Committee of The First People’s Hospital of Lin’an District (approval no. 2022-LA-THA-017). A total of 120 patients who underwent unilateral THA in the Department of Orthopedics between March 2022 and April 2025 were retrospectively included. According to the implementation timeline of different nursing protocols, patients treated from March 2022 to December 2023 received conventional analgesic care and were assigned to the control group (n = 60), while those treated from January 2024 to April 2025 received ERAS-based multimodal pain nursing and were assigned to the ERAS group (n = 60).

*Inclusion criteria were as follows*: Age ≥ 18 years; Primary unilateral elective THA; American Society of Anesthesiologists (ASA) physical status classification I–III; Complete clinical data with a minimum of 1-month postoperative follow-up.

*Exclusion criteria were as follows*: Severe cardiac, pulmonary, hepatic, or renal dysfunction; History of psychiatric disorders or cognitive impairment (e.g., Alzheimer disease, sequelae of cerebrovascular accidents); History of neuropathic pain (e.g., postherpetic neuralgia, diabetic peripheral neuropathy) or long-term analgesic use; Bilateral or revision THA; Intraoperative severe complications leading to interruption of the nursing protocol.

The study was reviewed and approved by the institutional ethics committee. Given the retrospective design, the requirement for informed consent was waived.

### 2.2. Study groups

According to the postoperative nursing protocols, the enrolled patients were divided into 2 groups, with 60 cases in each group.

The observation group received an ERAS-based multimodal pain nursing program. This protocol was implemented across 3 perioperative phases—preoperative, intraoperative, and postoperative—and emphasized coordinated, comprehensive strategies for pain control and functional recovery. The detailed interventions are described as follows:

#### 2.2.1. Preoperative phase

*Personalized education*: Operating room nurses, together with anesthesiologists, provided individualized education to patients. The content included an explanation of the ERAS concept, specific measures of multimodal pain management, and key points requiring patient cooperation. Case sharing was also incorporated to alleviate preoperative anxiety.

*Preemptive analgesia*: Patients received oral celecoxib (200 mg) 12 hours before surgery and oral acetaminophen (1000 mg) 2 hours before surgery, aiming to reduce the baseline level of preoperative pain.

#### 2.2.2. Intraoperative phase

*Nerve block*: Prior to the initiation of surgery, the anesthesiologist performed ultrasound-guided lumbar plexus block (0.5% ropivacaine, 20 mL) in combination with sciatic nerve block (0.5% ropivacaine, 15 mL).

*Local wound infiltration*: At the end of the procedure, the surgeon infiltrated the incision site with 0.25% ropivacaine (20 mL) to reduce postoperative incisional pain.

Intraoperative warming: A forced-air warming blanket was applied to maintain the patient’s body temperature between 36.0 and 37.0°C, in order to prevent hypothermia-induced stress response and heightened pain sensitivity.

#### 2.2.3. Postoperative phase

*Multimodal pharmacological analgesia*: At 6 hours postoperatively, patients received oral celecoxib (200 mg, twice daily) in combination with oral acetaminophen (1000 mg, 3 times daily). If the pain score was ≥4, dezocine (5 mg, intravenous injection) was administered as rescue analgesia.

*Dynamic pain assessment*: Pain was assessed using a combined approach of the numerical rating scale (NRS) and the behavioral pain scale. Assessments were performed every 2 hours within the first 24 hours postoperatively and every 4 hours thereafter, with the analgesic regimen adjusted according to the scores.

*Early rehabilitation guidance and psychological support*: At 6 hours postoperatively, patients were assisted with ankle flexion–extension exercises. At 12 hours, they were supported in turning over and sitting up. At 24 hours, patients were encouraged to stand or walk short distances with a walker, with activity intensity gradually increased according to tolerance.

*Control group (conventional analgesic care*): Patients received routine pain management. Specifically, 1 day prior to surgery, the responsible nurse provided education on pain-related knowledge (e.g., the impact of pain on recovery, pain assessment methods). Postoperatively, analgesia was administered according to physician orders based on patients’ pain complaints, using opioids (e.g., morphine, oxycodone) or nonsteroidal anti-inflammatory drugs (NSAIDs, such as ibuprofen, celecoxib), either orally or intravenously. Pain assessment was performed at fixed intervals (every 4–6 hours), and pain scores were documented.

### 2.3. Observational indicators

#### 2.3.1. Pain intensity

Postoperative pain was evaluated using the NRS at 6, 12, 24, 48, and 72 hours after surgery in both groups. The NRS ranges from 0 to 10, where 0 indicates no pain and 10 indicates the most severe pain. Scores of 0 to 3 represent mild pain, 4 to 6 moderate pain, and 7 to 10 severe pain.

#### 2.3.2. Rehabilitation progress and safety

*Rehabilitation indicators*: The time to first ambulation (defined as the time from the end of surgery to the first assisted standing with a walker), postoperative length of hospital stay (days from surgery to discharge), and hip function at 1 month postoperatively (assessed using the Harris hip score, total score = 100, covering pain, function, deformity, and range of motion, with higher scores indicating better hip function) were recorded. Analgesia-related indicator: The total opioid consumption within 48 hours after surgery was calculated and converted to morphine milligram equivalents (MMEs).

#### 2.3.3. Incidence of complications

Within 3 days postoperatively, adverse drug reactions (e.g., nausea, vomiting, dizziness, somnolence, respiratory depression, and constipation), incision-related complications (e.g., local erythema, tenderness), and rehabilitation-related complications (e.g., DVT, pulmonary infection) were recorded in both groups.

### 2.4. Statistical analysis

All statistical analyses were performed using SPSS Statistics version 26.0 (IBM Corp., Armonk, NY). A 2-sided significance level (α) of 0.05 was applied, and *P* < .05 was considered statistically significant. Continuous variables (e.g., age, body mass index (BMI), NRS pain scores, time to first ambulation, length of hospital stay, Harris hip score, and opioid consumption within 48 hours) were tested for normality using the Shapiro–Wilk test and for homogeneity of variance using Levene test. Normally distributed data with homogeneous variance were expressed as mean ± standard deviation $\left(\bar{x}\pm s\right)$ and compared between groups using the independent samples *t* test. Non-normally distributed data or those with unequal variances were expressed as median (interquartile range) and analyzed using the Mann–Whitney *U* test. Categorical variables (e.g., sex, ASA classification, comorbidities, and postoperative complications) were expressed as frequency and percentage [n (%)] and compared using the chi-square (*χ*^2^) or Fisher exact test, as appropriate. Ordinal data were analyzed using the Mann–Whitney *U* test. A post hoc power analysis based on the difference in mean NRS pain scores at 24 hours postoperatively yielded a statistical power (1−β) of 0.99, indicating adequate sample size to detect clinically meaningful differences between groups.

## 3. Results

### 3.1. Comparison of baseline characteristics between the 2 groups

This study retrospectively analyzed 120 patients who underwent primary unilateral THA. Based on their perioperative analgesic nursing regimen, patients were divided into 2 groups: the control group (n = 60), which received conventional analgesic care, and the observation group (n = 60), which received ERAS-based multimodal pain management. As shown in Table 1, a comprehensive comparison of baseline clinical characteristics was conducted between the 2 groups. The results demonstrated no statistically significant differences in age, sex, BMI, ASA classification, comorbidities (hypertension and diabetes mellitus), preoperative diagnosis, or preoperative Harris hip scores (all *P* > .05). Specifically, the mean age of patients in both groups was approximately 62 years, with a similar male proportion (control group: 46.7% vs observation group: 43.3%). The mean BMI was within the range of 23.2 to 23.5 kg/m^2^. ASA class II patients accounted for the majority in both groups (over 60%). The prevalence of hypertension and diabetes was also comparable between groups. The main preoperative diagnoses were osteonecrosis of the femoral head and osteoarthritis, with no significant difference in distribution. Moreover, preoperative Harris hip scores indicated similar levels of impaired hip function in both groups. In summary, the 2 groups were well balanced with respect to general demographic and clinical characteristics before surgery, thereby minimizing potential confounding factors and ensuring the reliability of subsequent comparative analyses of postoperative outcomes.

**Table 1:** Comparison of baseline characteristics between the 2 groups.

Variables	Control group (n = 60)	Observation group (n = 60)	*t*/*χ*^2^ value	*P* value
Age (yr, mean ± SD)	62.3 ± 5.8	61.8 ± 6.1	0.43	.67
Sex (male/female)	28/ 32	26/ 34	0.14	.71
BMI (kg/m^2^, mean ± SD)	23.5 ± 2.1	23.2 ± 2.3	0.61	.54
ASA classification (I/II/III)	10/38/12	11/37/12	0.05	.97
Hypertension [n (%)]	16 (26.7%)	15 (25.0%)	0.04	.84
Diabetes [n (%)]	5 (8.3%)	6 (10.0%)	0.08	.77
Preoperative diagnosis (ONFH/OA/FNF)*	22/ 20/ 18	21/ 19/ 20	0.12	.94
Preoperative Harris score (mean ± SD)	43.6 ± 6.7	44.1 ± 6.3	0.50	.62

* ASA = American Society of Anesthesiologists, BMI = body mass index, FNF = femoral neck fracture, OA = osteoarthritis, ONFH = osteonecrosis of the femoral head.

### 3.2. Comparison of postoperative NRS scores at different time points between the 2 groups

Compared with the control group, the NRS scores of patients in the observation group were significantly lower at 6, 12, 24, 48, and 72 hours postoperatively (all *P* < .05). As shown in Table 2, the overall trend indicates that postoperative pain scores decreased over time in both groups. However, pain control at each time point was superior in the observation group, suggesting that ERAS-based multimodal analgesia can more effectively reduce pain after total hip arthroplasty. The differences were most pronounced at 12 and 24 hours postoperatively, gradually diminishing thereafter, but remained statistically significant at 72 hours. This indicates that ERAS-based multimodal analgesia is particularly effective in alleviating early severe pain while maintaining analgesic advantages throughout the postoperative recovery period.

**Table 2:** Comparison of postoperative NRS scores between the 2 groups.

Time after surgery	Control group (n = 60)	Observation group (n = 60)	*t* value	*P* value
6 h	6.5 ± 1.0	3.5 ± 0.8	12.42	<.001
12 h	6.0 ± 1.1	2.8 ± 0.7	15.37	<.001
24 h	5.2 ± 1.0	2.5 ± 0.6	14.26	<.001
48 h	4.3 ± 0.9	2.2 ± 0.5	11.53	<.001
72 h	3.6 ± 0.8	2.0 ± 0.4	10.18	<.001

NRS = numerical rating scale.

### 3.3. Comparison of opioid consumption within 48 hours postoperatively between the 2 groups

The opioid consumption within 48 hours postoperatively differed significantly between the 2 groups, as shown in Table 3. The observation group consumed 39.6 ± 7.9 MMEs, which was significantly lower than the 52.4 ± 10.2 MMEs in the control group (*t* = 7.43, *P* < .001). These results indicate that multimodal analgesia can effectively reduce opioid requirements while maintaining adequate pain control.

**Table 3:** Comparison of opioid consumption within 48 h after surgery between the 2 groups.

Group	n	Opioid consumption (MMEs)	*t* value	*P* value
Control group	60	52.4 ± 10.2	7.43	<.001
Observation group	60	39.6 ± 7.9		

MMEs = morphine milligram equivalents.

### 3.4. Comparison of postoperative rehabilitation indicators between the 2 groups

The rehabilitation indicators of patients in the observation group were significantly better than those in the control group, as shown in Table 4. The time to first ambulation postoperatively was significantly earlier in the observation group compared with the control group (23.1 ± 5.0 hours vs 31.2 ± 6.3 hours, *t* = 7.64, *P* < .001). The length of postoperative hospital stay was significantly shorter in the observation group (7.3 ± 1.5 days vs 9.0 ± 1.7 days, *t* = 5.59, *P* < .001). The Harris hip score at 1 month postoperatively was significantly higher in the observation group (86.8 ± 5.5 vs 80.1 ± 6.0, *t* = 6.33, *P* < .001). All differences were statistically significant (*P* < .05). These findings indicate that ERAS-based multimodal analgesia not only improves pain control but also accelerates postoperative recovery and functional restoration.

**Table 4:** Comparison of postoperative recovery indicators between the 2 groups.

Indicator	Control group (n = 60)	Observation group (n = 60)	*t* value	*P* value
Time to first ambulation (h)	31.2 ± 6.3	23.1 ± 5.0	7.64	<.001
Length of hospital stay (d)	9.0 ± 1.7	7.3 ± 1.5	5.59	<.001
Harris hip score at 1 month	80.1 ± 6.0	86.8 ± 5.5	6.33	<.001

### 3.5. Comparison of postoperative complication rates between the 2 groups

The incidence of complications within 3 days postoperatively is shown in Table 5. The rate of drug-related adverse reactions in the observation group was 6.7%, significantly lower than 25.0% in the control group (*P* = .007), indicating that ERAS-based multimodal analgesia can reduce opioid use while effectively lowering the incidence of related adverse events. The incidence of incision-related complications was 3.3% in the observation group, also significantly lower than 15.0% in the control group (*P* = .028), suggesting that optimized analgesia helps alleviate incision discomfort. Regarding rehabilitation-related complications, the incidence was 1.7% in the observation group versus 6.7% in the control group; although a decreasing trend was observed, the difference was not statistically significant (*P* = .362). Overall, the total complication rate was 11.7% in the observation group, significantly lower than 46.7% in the control group (*P* < .001), indicating that multimodal analgesia can not only ensure effective pain control and promote recovery but also significantly reduce the overall risk of early postoperative complications.

**Table 5:** Comparison of postoperative complications within 3 days between the 2 groups.

Complication type	Observation group (n = 60)	Control group (n = 60)	*χ*^2^ value	*P* value
Adverse drug reactions	4 (6.7%)	15 (25.0%)	7.273	.007
Nausea, vomiting, dizziness	4 (6.7%)	15 (25.0%)		
Incisional pain-related complications	2 (3.3%)	9 (15.0%)	4.800	.028
Redness, tenderness	2 (3.3%)	9 (15.0%)		
Rehabilitation-related complications	1 (1.7%)	4 (6.7%)	–	.362*
Deep vein thrombosis	0 (0.0%)	2 (3.3%)		
Pulmonary infection	1 (1.7%)	2 (3.3%)		
Total complications	7 (11.7%)	28 (46.7%)	14.860	<.001

## 4. Discussion

This study retrospectively compared the effects of ERAS-based multimodal analgesia versus conventional pain management in patients undergoing THA. The results demonstrated that multimodal analgesia not only significantly reduced NRS pain scores during the early postoperative period (6, 12, 24, 48, and 72 hours) but also decreased opioid consumption within 48 hours postoperatively, facilitated early mobilization, shortened the length of hospital stay, and resulted in higher Harris hip scores at 1 month after surgery. Moreover, the incidence of postoperative complications in the observation group was significantly lower than that in the control group, indicating that multimodal analgesia can safely and effectively enhance patient recovery.

In terms of pain management, our findings are consistent with previous studies, showing that ERAS-based multimodal analgesia significantly alleviates postoperative pain following THA.^[15]^ Although conventional opioid-based analgesia provides effective pain relief, its high incidence of adverse effects—such as nausea, vomiting, and dizziness—often compromises patient adherence to rehabilitation. In contrast, multimodal analgesia combines nonsteroidal anti-inflammatory drugs, local infiltration analgesia, nerve blocks, and non-pharmacological interventions (e.g., psychological support and positioning care). By leveraging complementary mechanisms of action, this approach reduces opioid consumption and thereby lowers the risk of drug-related adverse events. Importantly, in this study, the opioid consumption within the first 48 hours postoperatively was significantly lower in the observation group than in the control group. This “opioid-sparing effect” has important clinical implications, directly leading to a significant reduction in opioid-related adverse events (e.g., nausea and vomiting), consistent with the findings of Khellah colleagues.^[16–18]^ Fundamentally, this approach improves the perioperative experience and enhances patient safety, further validating the efficacy and safety of the multimodal analgesia strategy.

Secondly, regarding rehabilitation outcomes, the results of this study indicate that patients receiving multimodal analgesia experienced an accelerated postoperative recovery. The time to first ambulation in the observation group was significantly earlier than that in the control group. Early mobilization is a critical component of the ERAS pathway, as it not only effectively reduces the risk of bed-related complications such as lower extremity DVT and pulmonary infections, but also promotes muscle strength recovery and prevents joint stiffness, thereby establishing a positive recovery cycle.^[19,20]^ Benefiting from effective pain control and early mobilization, patients in the observation group had significantly shorter postoperative hospital stays, which not only reduced healthcare resource utilization but also lowered the risk of hospital-acquired infections.^[21]^ More importantly, effective functional exercises under early pain-free conditions yielded long-term functional benefits. The Harris hip score at 1 month postoperatively was significantly higher in the observation group compared with the control group, indicating that optimal postoperative pain management creates favorable conditions for rehabilitation and accelerates overall recovery.^[22]^

Furthermore, regarding the incidence of complications, this study showed that patients in the observation group experienced significantly lower rates of drug-related adverse reactions and incision-related complications compared with the control group. Due to the limited sample size, no statistical differences were observed for certain individual indicators such as DVT; however, there was an overall downward trend in rehabilitation-related complications in the observation group, and the difference in total complication rate was statistically significant. This is not only closely related to the reduction in opioid use achieved through multimodal analgesia, but may also be associated with improved patient compliance and the smooth implementation of early rehabilitation exercises following effective pain relief, suggesting that multimodal care may have potential advantages in preventing such complications.^[23]^

In addition, the feasibility and challenges of implementing ERAS-based multimodal pain nursing at different medical levels deserve further consideration. In tertiary hospitals, the availability of multidisciplinary teams and standardized clinical pathways facilitates the integration of ERAS protocols. However, in secondary and primary care institutions, limited resources, insufficient staff training, and inconsistent awareness of ERAS concepts may hinder widespread adoption. To promote broader implementation, it is necessary to strengthen professional education for nurses and anesthesiologists, establish unified nursing standards, and improve interdisciplinary collaboration mechanisms. Future studies should also evaluate cost-effectiveness and patient satisfaction to provide practical evidence for large-scale application of ERAS multimodal care in diverse healthcare settings.

However, this study has several limitations. First, as a single-center retrospective study, it is inherently limited by potential selection bias, information bias, and incomplete control of confounding factors. The retrospective nature also restricted the ability to standardize all perioperative variables and to establish causal relationships. Second, the relatively small sample size may limit the statistical power to detect subtle intergroup differences and reduce the generalizability of the findings. Although baseline characteristics between groups were comparable, potential confounding factors—such as surgical approach, anesthesia method, intraoperative blood loss, and surgeon experience—could not be entirely excluded. No multivariate regression adjustment was performed because perioperative management protocols were largely standardized within the institution. Third, the follow-up duration was short, focusing only on outcomes within 1 month postoperatively, which may not fully capture long-term functional recovery, chronic pain outcomes, or late complications. Therefore, future multicenter, large-sample, prospective randomized controlled studies with extended follow-up periods are needed to validate and expand upon the present findings.

In conclusion, ERAS-based multimodal analgesia can effectively alleviate postoperative pain following THA, reduce opioid consumption, promote early rehabilitation, and lower the incidence of complications, demonstrating substantial clinical applicability. The results of this study provide strong evidence for optimizing postoperative pain management strategies in THA and support the implementation of the ERAS concept in orthopedic perioperative care.

## Author contributions

**Conceptualization:** Yumei Fang, Yixin Yu.

**Data curation:** Yumei Fang, Yixin Yu.

**Formal analysis:** Yumei Fang, Yixin Yu.

**Funding acquisition:** Yumei Fang, Yixin Yu.

**Investigation:** Yumei Fang, Yixin Yu.

**Writing – original draft:** Yumei Fang, Yixin Yu.

**Writing – review & editing:** Yumei Fang, Yixin Yu.
